# How Family and Individual Physical Activity Environments Relate to Obesity Transition in Chinese Children and Adolescents

**DOI:** 10.3390/nu17233760

**Published:** 2025-11-29

**Authors:** Ziyue Sun, Yang Yang, Xia Zhong, Jiajia Dang, Shan Cai, Yunfei Liu, Jiaxin Li, Tianyu Huang, Xiaoqian Zhang, Mei Xue, Jing Li, Zhixin Zhang, Yi Song

**Affiliations:** 1 Institute of Child and Adolescent Health, School of Public Health, Peking University, Beijing 100191, China; sunziyue@bjmu.edu.cn (Z.S.); 2516395133@bjmu.edu.cn (Y.Y.); zhongxia-pku@bjmu.edu.cn (X.Z.); dangjj@bjmu.edu.cn (J.D.); 1710306207@pku.edu.cn (S.C.); melatonin@pku.edu.cn (Y.L.); lijiaxin@bjmu.edu.cn (J.L.); tyhuang@pku.edu.cn (T.H.); 2 National Health Commission Key Laboratory of Reproductive Health, Beijing 100191, China; 3 Department of Traditional Chinese Medicine, Beijing Children’s Hospital, Capital Medicine University, National Center for Children’s Health, Beijing 100045, China; 18661312368@163.com; 4 Graduate School, Beijing University of Chinese Medicine, Beijing 100105, China; xuemei202407@163.com; 5 Department of Pediatrics, China-Japan Friendship Hospital, Beijing 100029, China; 6 Key Laboratory of Epidemiology of Major Diseases Peking University, Ministry of Education, Beijing 100191, China; 7 Institute of Clinical Medical Sciences, China-Japan Friendship Hospital, Beijing 100029, China

**Keywords:** physical activity environment, overweight, obesity, children and adolescents

## Abstract

**Background**: Overweight and obesity have emerged as major public health challenges in China, with rising prevalence rates posing substantial burdens on healthcare systems. This study is a secondary analysis of data from the 2019 Chinese National Survey on Students’ Constitution and Health (CNSSCH), aimed to investigate the association of family and individual physical activity (PA) environments with overweight and obesity transition among children and adolescents, and to analyze subgroup differences by sex and urban–rural location as well as potential mechanisms. **Methods**: This was a one-year longitudinal study based on the 2019–2020 follow-up of 5008 children and adolescents. Family and individual physical activity environments were assessed as main exposures, and transitions to overweight and obesity were defined as outcomes. Multivariate logistic regression was applied to evaluate the association between family and individual PA environments and transitions to overweight and obesity, while also examining their moderating effects on unhealthy lifestyle behaviors. **Results**: A favorable family and individual PA environment was significantly associated with a reduced risk of obesity transition (OR = 0.78, *p* = 0.016). Subgroup analyses indicated that males (OR = 0.69, *p* = 0.009) and rural children and adolescents (OR = 0.70, *p* = 0.021) were more sensitive to supportive family and individual PA environments. Furthermore, a favorable environment was significantly correlated with a reduction in unhealthy behaviors such as skipping daily breakfast and consuming sugar-sweetened beverages (*p* < 0.001). **Conclusions**: A supportive family and individual PA environment was associated with a lower risk of obesity transition in children and adolescents, with more pronounced associations observed among males and those in rural areas.

## 1. Introduction

The escalating prevalence of childhood and adolescent obesity is reshaping the global public health landscape. According to the World Health Organization, the prevalence of overweight and obesity among children and adolescents aged 5–19 years has reached 20% [1], indicating that nearly one in five faces obesity-related health risks, including early-onset hypertension, insulin resistance, and psychosocial problems [2,3,4]. In China, rapid economic growth and lifestyle modernization have driven a more than twofold increase in overweight and obesity rates over the past two decades. This trend not only heightens healthcare burdens but also highlights the urgency of identifying effective intervention strategies. Obesity arises from the interplay of genetic, behavioral, and environmental determinants [5,6,7]. Among these, environmental factors, particularly family and individual physical activity (PA) environments, are increasingly recognized as highly modifiable and promising targets for intervention [8].

The family PA environment serves as a cornerstone. It is reflected not only in parents’ regular exercise behaviors but also in the support they provide for their children’s activity, such as encouraging participation in extracurricular sports, supplying equipment, or organizing family outings [8]. Through role modeling and emotional support, as framed by social learning theory, these practices profoundly shape children’s exercise habits [9,10]. By contrast, the individual PA environment emphasizes intrinsic motivation. For example, a child’s passion for basketball drives daily practice or a self-challenge that inspires goal-setting in running [11]. Such intrinsic factors largely determine the sustainability and depth of physical activity, aligning with self-determination theory, which underscores the importance of autonomy and interest [12,13].

Overweight and obesity are distinct but progressive conditions defined by age- and sex-specific BMI percentiles. Biologically, overweight reflects mild energy imbalance and adipose tissue accumulation, with limited metabolic disturbances [14]. Obesity involves excessive adipose tissue expansion, leading to chronic low-grade inflammation, insulin resistance, and increased risk of comorbidities [15]. The transition from normal to overweight or obesity is driven by sustained positive energy balance, where behavioral factors interact with environmental cues to accelerate adipose tissue growth [16]. Clinically, intervening during the pre-obesity (overweight) stage is more effective for weight reversal, while obesity transition marks a critical point where metabolic adaptations make weight management more challenging [17].

A study of Spanish families reported that greater parental engagement in PA was associated with lower risk of childhood overweight [18], while an international meta-analysis demonstrated that enjoyment of exercise strongly predicts long-term weight management success [19]. Current research on PA environments and childhood obesity has two key gaps. First, most studies focus on either family support or individual motivation in isolation: cross-sectional studies have linked parental PA participation to lower childhood overweight risk [18], while individual intrinsic motivation is associated with long-term weight management [19], but few have explored their synergistic effect on obesity transition. Second, sex and urban-rural disparities remain underexplored: male adolescents may be more responsive to family PA role models due to sociocultural norms [20], while rural children may rely more on family support due to limited community PA resources [21], but these heterogeneities lack empirical validation in longitudinal studies. Third, the mediating role of unhealthy behaviors in linking PA environments to obesity transition has not been fully verified. Based on these gaps, we hypothesized that: (1) a favorable combined family and individual PA environment is associated with a reduced risk of obesity transition; (2) this association varies by sex and urban-rural residence; and (3) unhealthy lifestyle behaviors mediate this association.

## 2. Materials and Methods

### 2.1. Study Design and Participants

**Figure 1:**
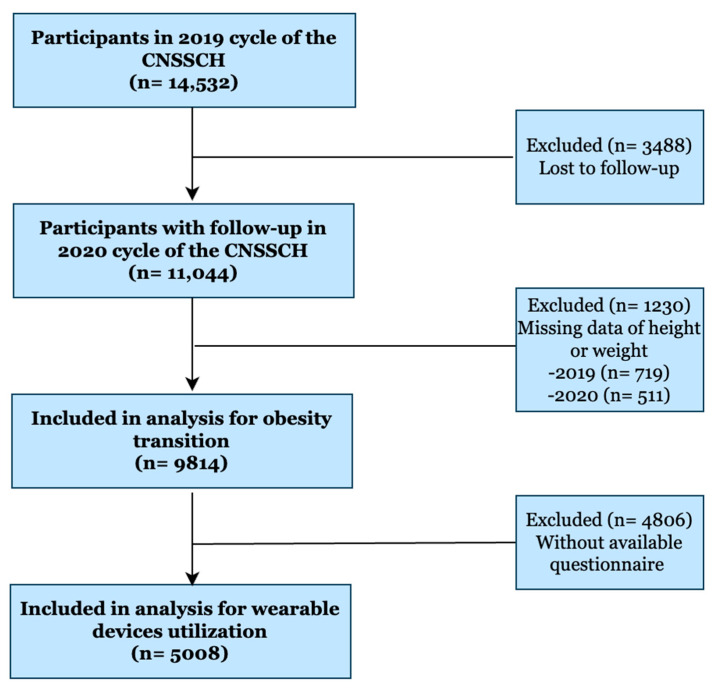
Study flow.

This longitudinal analysis utilized baseline data from the 2019 Chinese National Survey on Students’ Constitution and Health (CNSSCH) [22]. A stratified random sampling method was employed, selecting eight provinces representing eastern, central, and western regions (Shanghai, Fujian, Shanxi, Henan, Hunan, Gansu, Chongqing, and Guangxi) for follow-up assessment conducted in November 2020. This study involved a 12-month follow-up period from November 2019 to November 2020. Of the 14,532 adolescents aged 9–18 years in the baseline cohort (Figure 1), 9814 participants who met the age criteria were included in subsequent analyses. The study flow diagram was constructed in accordance with the STROBE (Strengthening the Reporting of Observational Studies in Epidemiology) guidelines [23]. The final analytical sample comprised 5008 respondents who completed the full questionnaire. The reduction in sample size was due to missing questionnaires or inability to reach certain students during the school-based follow-up visit. The study protocol was approved by the Peking University Institutional Review Board (IRB00001052-18002, IRB00001052-21001).

### 2.2. Measurement of Overweight, Obesity, and Transitions

Anthropometric assessments were conducted by trained staff following standardized procedures. Participants wore light clothing and were measured barefoot. Height and weight were recorded to the nearest 0.1 cm and 0.1 kg, respectively. Body mass index (BMI) was calculated as weight (kg)/height (m)^2^, and weight status was classified according to age- and sex-specific BMI percentiles issued by the National Health Commission of China [24]: overweight (OW) ≥ 85th percentile, obesity (OB) ≥ 95th percentile. This standard is tailored to the growth and development characteristics of Chinese children and adolescents, which differ from Western populations in terms of body composition and growth trajectories. The aforementioned sensitivity analysis indirectly supports comparability with International OB Task Force (IOTF), as the core findings remained unchanged when alternative, internationally recognized cut-offs were applied [25]. Overweight transition was defined as a shift from normal weight or underweight at baseline to overweight at follow-up, while obesity transition was defined as a shift from normal weight, underweight, or overweight at baseline to obesity at follow-up, based on the Chinese national criteria [24].

### 2.3. Assessment of Family and Individual Physical Activity Environments

The family PA environment was evaluated using two questions: (1) “Do your parents support you in engaging in physical activity during your spare time?” (responses: “strongly support,” “support,” “neutral,” “rarely support,” coded as 3, 2, 1, and 0 points, respectively); and (2) “Do your parents enjoy participating in physical activity during their leisure time?” (responses: “both parents,” “father only,” “mother only,” “neither parent,” coded as 2, 1, 1, and 0 points, respectively). The total family PA environment score ranged from 0 to 5, with scores ≥ 3 defined as “favorable” and <3 as “unfavorable.”

The individual PA environment was assessed using nine items: physical activity makes me look better; gives me more energy; makes me feel happier; is enjoyable; helps me make more friends; makes me stronger; makes me like myself more; improves my body shape; and makes me feel healthier. Each item was rated on a 5-point Likert scale (“strongly agree,” “agree,” “uncertain,” “disagree,” “strongly disagree”), scored as 3, 2, 1, 0, and 0 points, respectively. The total individual PA environment score ranged from 0 to 27, with scores ≥ 19 defined as “favorable” and <19 as “unfavorable”.

The family PA environment scale has a total score range of 0–5. The midpoint is 2.5; we rounded up to 3, defining scores ≥ 3 as “favorable” and <3 as “unfavorable.” This midpoint-based division is intuitive, ensuring clear differentiation between supportive and non-supportive family environments. The individual PA environment scale has a total score range of 0–27. The midpoint is 13.5; we set ≥19 as “favorable” (exceeding the midpoint by ~40%) to reflect a meaningful level of intrinsic motivation for PA. This adjustment was made because intrinsic motivation requires a higher degree of positive perception (vs. mere “neutral” attitudes) to drive sustained behavior. The combined PA environment score was calculated as the sum of family and individual scores (range 0–32). A total score ≥23 was defined as “favorable” and <23 as “unfavorable.” This aligns with the component scales: it represents the sum of the family threshold (3) and the individual threshold (19), ensuring consistency across assessments. The threshold setting was further validated by referencing a well-cited study on school PA environment assessment [24], which adopted a midpoint-based dichotomization method for its scale (total score 0–40, midpoint 20, threshold ≥20 for “favorable”).

### 2.4. Questionnaire Survey

Our study utilized the CNSSCH questionnaire, a standardized and nationally validated tool administered across China every five years. The full name of the questionnaire is: “2019 National Survey on Students’ Constitution and Health Questionnaire”, authored by the Working Group of the Chinese National Survey on Students’ Constitution and Health. This questionnaire is widely used in national epidemiological surveillance of child and adolescent health and includes structured modules on physical activity, family lifestyle habits, diet, and health behaviors. The questionnaire was administered in Mandarin Chinese, the standard language of instruction in Chinese schools. It has been shown to have satisfactory psychometric properties; specifically, prior validation studies reported moderate internal consistency, with a Cronbach’s alpha of 0.76 for school-level behavioral and environmental factors [26]. All items used in our study were drawn directly from this validated national instrument, with no additional modifications. The survey was self-completed under researcher supervision, with clarifications provided when necessary. Information collected included: (1) sociodemographic characteristics (age, sex, residence, grade level, only-child status, and parental education); and (2) health-related behaviors (sugar-sweetened beverage intake frequency, daily sleep patterns, and physical activity participation) [26].

### 2.5. Statistical Analysis

Missing values in covariates and questionnaire items were imputed using a hot-deck approach [27]. Donors were selected from participants within the same province and of the same sex and age group (±1 year). For each recipient case with missing data, a donor record with complete information was randomly drawn from the donor pool and used to fill missing items; five imputed datasets were created and combined using Rubin’s rules for variance estimation. Differences in categorical variables across sex and residence were tested using the χ^2^ test, while age-related trends were assessed using the Mantel–Haenszel χ^2^ test for linear trend. Multilevel logistic regression models were applied to estimate adjusted odds ratios (ORs) and 95% confidence intervals (CIs) for behavioral predictors of overweight/obesity incidence and transitions, incorporating province-level random intercepts to account for the hierarchical data structure. Candidate confounders were selected a priori based on existing literature and causal diagrams (DAG), and included age, sex, urban/rural residence, only-child status, parental education level, breakfast frequency, sugar-sweetened beverage intake, and physical activity time. We assessed multicollinearity by variance inflation factors (VIF) and retained covariates with VIF < 5. Effect modification by sex and residence was tested by including multiplicative interaction terms and by conducting stratified analyses. All analyses were performed using SPSS version 26.0 and R version 4.2.2, with two-tailed tests and a significance level set at α = 0.05.

## 3. Results

### 3.1. Association Between Family and Individual Physical Activity Environments and Obesity Transitions

A total of 5008 children and adolescents were included in the analysis, comprising 2508 boys (50.1%) and 2500 girls (49.9%) (Table 1). Differences in categorical variables across sex and residence were tested using the χ^2^ test, while age-related trends were assessed using the Mantel–Haenszel χ^2^ test for linear trend. The incidence of overweight and obesity was 14.3% and 11.3%, respectively. Specifically, 6.2% of participants transitioned from normal weight to overweight, and 10.1% transitioned to obesity. Regarding family and individual physical activity (PA) environments, 45.6% of children and adolescents were classified as being in an unfavorable environment, while 54.4% were in a favorable environment (*p* < 0.001). Among these, 76.1% of participants had a favorable family PA environment, and 57.0% had a favorable individual PA environment, both showing significant sex differences.

Further examination of the indicators used to assess the family PA environment revealed that 41.9% of parents strongly supported their children’s participation in extracurricular PA, and 48.1% of parents themselves engaged in PA during leisure time. For individual PA environment indicators, more than half of participants agreed that “physical activity gives me more energy” (48.8%) and “physical activity makes me happier” (46.6%), while 44.2% endorsed that “physical activity is enjoyable.” Additionally, 40.0% reported that PA helped them make more friends, 57.7% believed it made them stronger, 37.5% felt it increased self-liking, 45.0% agreed it improved body shape, and 67.1% stated it made them feel healthier.

**Figure 2:**
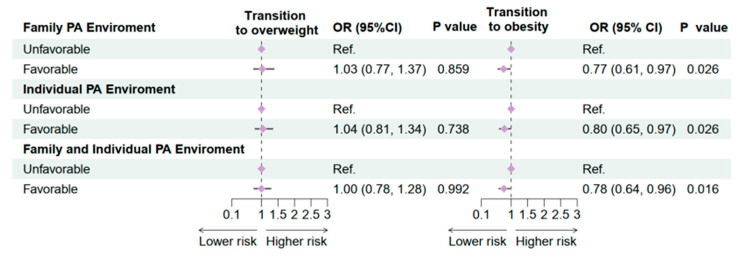
Association Between family and individual physical activity environment and OWOB transition in 2019–2020 follow-up study. OR: Odds ratio; CI: Confidence interval. PA: Physical activity. OW: Overweight; OB: Obesity; Adjusted for age, sex, residence, single-child status, breakfast frequency, sugar-sweetened beverage intake, sleeping duration, parental education level, and the clustered effect of provinces.

We further assessed the associations between family and individual PA environments and transitions to overweight and obesity (Figure 2). Overweight transition was defined as a shift from normal weight or underweight at baseline to overweight at follow-up, while obesity transition was defined as a shift from normal weight, underweight, or overweight at baseline to obesity at follow-up, based on the Chinese national criteria [24]. Results showed that a favorable family PA environment was not significantly associated with overweight transition (OR = 1.03, 95% CI: 0.77–1.37, *p* = 0.859), but was significantly associated with reduced risk of obesity transition (OR = 0.77, 95% CI: 0.61–0.97, *p* = 0.026). Similarly, a favorable individual PA environment was not significantly associated with overweight transition (OR = 1.04, 95% CI: 0.81–1.34, *p* = 0.738), but was significantly protective against obesity transition (OR = 0.80, 95% CI: 0.65–0.97, *p* = 0.026). When both family and individual PA environments were considered together, no significant association was observed with overweight transition (OR = 1.00, 95% CI: 0.78–1.28, *p* = 0.992), whereas a significant protective effect was observed for obesity transition (OR = 0.78, 95% CI: 0.64–0.96, *p* = 0.016). These findings suggest that favorable family and individual PA environments can effectively reduce the risk of transitioning from normal weight to obesity among children and adolescents.

### 3.2. Subgroup Differences in the Association Between Family and Individual Physical Activity Environments and Obesity Transitions

**Figure 3:**
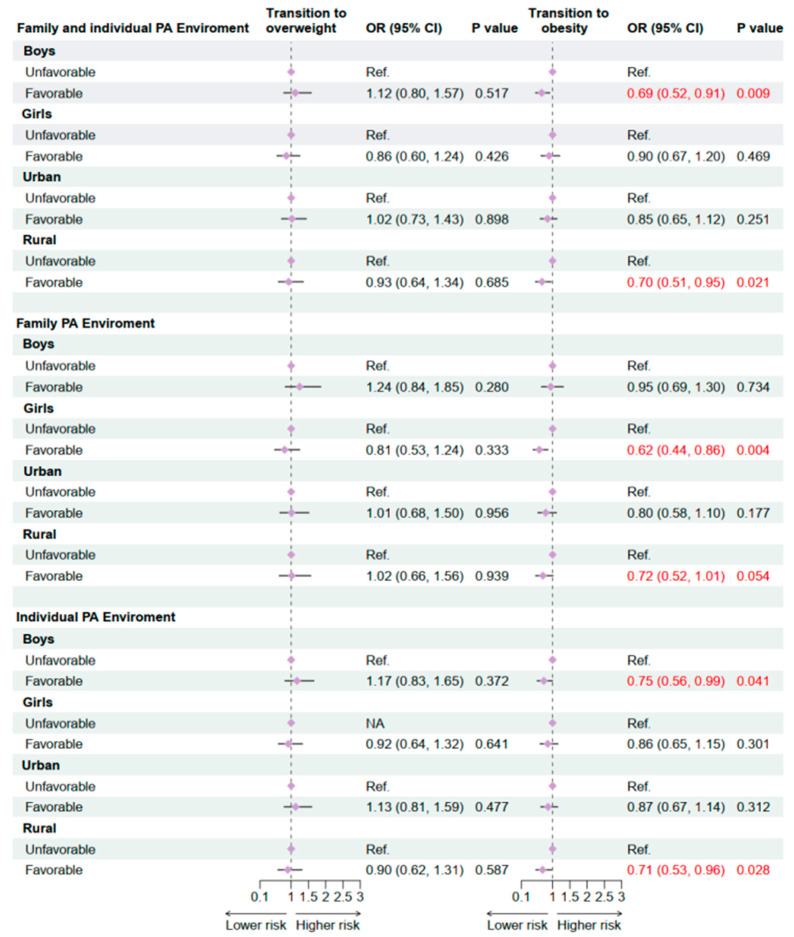
Association Between family and individual physical activity environment and OWOB transition in 2019–2020 follow-up study by subgroups. OR: Odds ratio; CI: Confidence interval. PA: Physical activity. OW: Overweight; OB: Obesity; Adjusted for age, sex, residence, single-child status, breakfast frequency, sugar-sweetened beverage intake, sleeping duration, parental education level, and the clustered effect of provinces. The red font in the figure is used to highlight the statistically significant differences.

Within family and individual PA environments, a significant inverse association with obesity transition was observed among males (OR = 0.69, 95% CI: 0.52–0.91, *p* = 0.009) (Figure 3), indicating that favorable environments reduced the risk of transitioning to obesity. No similar association was found among females (OR = 0.90, 95% CI: 0.67–1.20, *p* = 0.469). When family and individual PA environments were analyzed separately, a favorable family PA environment showed a stronger protective association among females (OR = 0.62, 95% CI: 0.44–0.86, *p* = 0.004), whereas a favorable individual PA environment demonstrated significant protection among males (OR = 0.75, 95% CI: 0.56–0.99, *p* = 0.041) but not females (OR = 0.86, 95% CI: 0.65–1.15, *p* = 0.301). Overall, these findings suggest that males may be more sensitive to favorable features of family and individual PA environments, and optimizing such environments may yield greater benefits for obesity prevention among boys.

In urban–rural stratified analyses, children and adolescents in rural areas exhibited stronger sensitivity to PA environments. A favorable family and individual PA environment was significantly associated with a reduced risk of obesity transition in rural participants (OR = 0.70, 95% CI: 0.51–0.95, *p* = 0.021) (Figure 3). For the family PA environment dimension alone, the association approached significance (OR = 0.72, 95% CI: 0.52–1.01, *p* = 0.054), suggesting a potential protective trend. In contrast, no significant associations were observed in urban participants across any environmental dimension. Taken together, these results indicate that the influence of family and individual PA environments on obesity transitions varies by sex and residence. Male and rural adolescents appear more responsive to favorable PA environments, highlighting these groups as priority targets for environment-based obesity prevention strategies.

### 3.3. Association Between Family and Individual Physical Activity Environments and Unhealthy Behaviors

**Figure 4:**
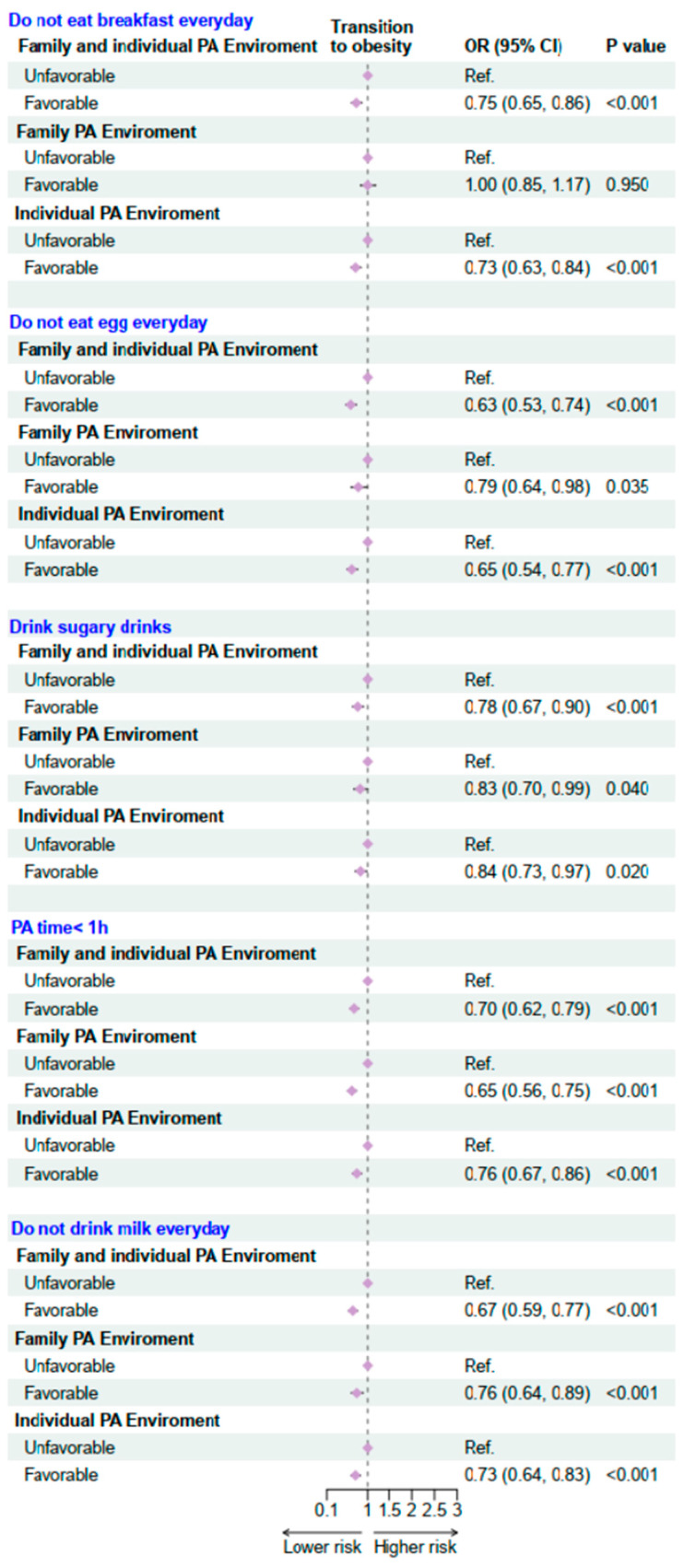
Association Between family and individual physical activity environment and risk factors in 2019–2020 follow-up study. OR: Odds ratio; CI: Confidence interval. PA: Physical activity. Adjusted for age, sex, residence, single-child status, breakfast frequency, sugar-sweetened beverage intake, sleeping duration, parental education level, and the clustered effect of provinces.

Regarding the unhealthy behavior of skipping breakfast, a favorable family and individual physical activity environment was significantly associated with a reduced risk of not eating breakfast daily (OR = 0.75, 95% CI: 0.65–0.86, *p* < 0.001) (Figure 4). The strongest protective effect was observed for not consuming eggs daily (OR = 0.63, 95% CI: 0.53–0.74, *p* < 0.001). In terms of sugar-sweetened beverage consumption, a favorable family and individual PA environment jointly exerted a protective effect, significantly lowering the likelihood of this unhealthy behavior (OR = 0.78, 95% CI: 0.67–0.90, *p* < 0.001). Similarly, the presence of a favorable PA environment significantly reduced the risk of insufficient physical activity (OR = 0.70, 95% CI: 0.62–0.79, *p* < 0.001). For not drinking milk daily, a protective association was also evident (OR = 0.67, 95% CI: 0.59–0.77, *p* < 0.001). Overall, these findings suggest that favorable family and individual PA environments confer substantial protective effects against a range of unhealthy lifestyle behaviors among children and adolescents.

## 4. Discussion

This study, based on a nationwide longitudinal cohort covering eight provinces in eastern, central, and western China, included 5008 children and adolescents aged 9–18 years. It systematically examined the association between family and individual PA environments and the transition to obesity, while further exploring subgroup differences and potential mechanisms. We found that a favorable combined family and individual PA environment was significantly associated with a reduced risk of obesity transition (OR = 0.78, *p* = 0.016). The protective effect of an optimized environment was particularly evident during the critical stage when body weight progresses toward obesity. Subgroup analyses revealed substantial heterogeneity: the protective effect of a favorable PA environment was more pronounced among males (OR = 0.69, *p* = 0.009) and rural children (OR = 0.70, *p* = 0.021), whereas no significant associations were observed among females or urban populations. These findings provide population-specific evidence for precision-targeted interventions. Furthermore, favorable PA environments significantly attenuated several unhealthy lifestyle behaviors, including skipping breakfast (OR = 0.75, *p* < 0.001), sugar-sweetened beverage consumption (OR = 0.78, *p* < 0.001), and insufficient physical activity (OR = 0.70, *p* < 0.001), suggesting that such behaviors may act as potential mediators linking PA environments to obesity transition through the dual regulatory pathway.

The protective effect of the family and individual PA environment identified in our study (OR = 0.78, *p* = 0.016) is consistent with, but substantially stronger than, previous longitudinal findings. For instance, Hobbs et al. (2019) reported a more modest protective association focusing specifically on the availability of PA facilities and parks (OR = 0.979) [28]. However, their study primarily emphasized PA facilities and parks, without accounting for individual PA motivation or the broader family PA environment. By integrating both family support and individual intrinsic motivation, the present study is the first to demonstrate their synergistic effect in attenuating obesity progression. Moreover, the protective role of individual PA environments on long-term weight management corroborates findings by Elsborg et al. (2018), which showed that individuals with strong PA interest had a higher success rate in weight control [29]. Our study further refined this evidence, showing that such an effect was restricted to the obesity transition but not the overweight transition. A plausible explanation is that during the overweight stage, energy imbalance is relatively mild, and environmental improvements may not suffice to reverse weight trajectories; whereas approaching the obesity threshold, enhanced PA environments may play a decisive role by regulating lifestyle behaviors and increasing energy expenditure.

Sex differences revealed in this study extend beyond previously documented disparities in PA participation rates. Our findings that males were more sensitive to PA environments may be partly explained by sociocultural expectations in China: traditional norms emphasize that “boys should be more active,” leading families to provide more direct support for male children [30]. Conversely, females may experience diminished effects from PA environments due to body image concerns and a lack of female-friendly PA programs [31,32]. These differences are not dominated by life events or educational disparities, but rather by inherent biological differences and socialization processes. Regarding urban–rural disparities, the pronounced protective effect in rural populations and the absence of associations in urban settings may stem from unequal distribution of PA resources [33]. Rural children often rely predominantly on family-driven PA due to limited access to community facilities and school playgrounds, making family and individual PA environments critical for weight management [34]. In contrast, urban children benefit from diverse opportunities through extracurricular sports clubs and public facilities, diluting the relative influence of family and individual environments. However, resource accessibility remains the core driver, as even with similar educational inputs, rural children’s PA opportunities are constrained by environmental limitations.

The observed associations can be explained by two theoretical frameworks and the mediating role of unhealthy behaviors. Firstly, according to Social Learning Theory, the family PA environment serves as a “behavioral demonstration field”: when parents engage in leisure-time exercise or support their child’s PA participation, they provide observable and replicable behavior models [35]. Children, through processes of observation, imitation, and reinforcement, establish stable exercise habits [36]. This mechanism may be particularly critical in rural areas, where external role models such as coaches or sports clubs are scarce, making the family the primary carrier of PA behavior transmission [37]. Secondly, from the perspective of Self-Determination Theory, the individual PA environment reflects intrinsic motivation [38]. When children derive joy, confidence, or social fulfillment from PA, their participation becomes autonomous rather than externally imposed, thereby enhancing both persistence and intensity [39]. For males, this intrinsic motivation aligns with cultural expectations of “male vitality,” further reinforcing exercise habits and reducing obesity risk [40]. Finally, the mediating role of unhealthy behaviors constitutes a key pathway linking PA environments to obesity transition. Favorable PA environments promote regular eating behaviors, suppress high-sugar intake, and enhance PA participation, collectively achieving a balanced regulation of energy intake and expenditure. For instance, children in supportive PA environments may be more likely to maintain breakfast consumption to meet energy demands for exercise, consciously reduce sugary drinks due to enhanced health awareness from PA, and simultaneously increase energy expenditure through regular exercise, thereby mitigating the risk of progression to obesity.

This study has several limitations. First, the follow-up period was only one year, which restricts our ability to examine long-term patterns. Second, PA environments were measured using self-reported items and may be subject to reporting bias. Third, despite adjusting for multiple confounders, residual confounding cannot be ruled out. Finally, family structure and parental occupation may influence the availability of family PA support and children’s PA opportunities. Due to data limitations in the secondary analysis, these variables were not included in the current model. Future studies should collect data on family structure, parental occupation, and work hours to explore how these factors interact with family PA environments to affect obesity transition.

Our study contributes to achieving Sustainable Development Goals (SDG 3: Good Health and Well-being; SDG 10: Reduced Inequalities) by identifying modifiable environmental factors to mitigate childhood obesity and address urban-rural/sex disparities in weight management. Family physical activity environments are highly modifiable through targeted public health interventions, as they are influenced by behavioral, social, and policy-related factors. In China, the “Healthy China 2030” initiative highlights family-centered health promotion, offering a supportive policy framework for scaling such interventions. This is particularly relevant in rural areas, where the family PA environment demonstrates a stronger protective effect and may benefit from low-cost, high-impact strategies—such as providing community-based PA equipment combined with family-oriented activity challenges. To effectively promote family PA, school-family collaborative programs could be introduced, such as “Parent-Child PA Challenges” facilitated through schools. These campaigns could allow families to track joint activities via WeChat mini-programs, with schools offering incentives for consistent participation to encourage sustained engagement through social influence. Additionally, a user-friendly mobile application could be developed to provide personalized PA recommendations, family activity logging, and educational content on the benefits of regular exercise. At the community level, nudge strategies—such as displaying success stories of active families on bulletin boards and social media—could strengthen social proof. Prompts and signage in parks and public spaces could also encourage spontaneous physical activity. Finally, integrating a “family PA support rate” as a key metric in local health performance evaluations could institutionalize such efforts. Linking this indicator to funding incentives for schools and communities would further enhance motivation and accountability, ensuring lasting commitment across multiple levels.

## 5. Conclusions

This study examined the associations and potential mechanisms between family and individual PA environments and obesity transition among children and adolescents. Our findings suggest that supportive family and individual PA environments may be associated with a lower short-term risk of transitioning to obesity. Subgroup analyses further revealed population heterogeneity, with boys and rural adolescents being more sensitive to the protective effects of favorable PA environments. Mechanistically, favorable PA environments may exert their influence by curbing unhealthy lifestyle behaviors, thereby interrupting the progression to obesity. In summary, this study provides evidence of family and individual PA environments in obesity transition among children and adolescents, identifies boys and rural populations as key target groups for PA environment optimization, and provides scientific evidence to inform targeted prevention strategies that support precision obesity control in youth. However, these associations should be interpreted with caution due to the one-year follow-up period. Future studies with longer follow-up durations and objective measures of physical activity are needed to validate these findings and clarify causal pathways.

## Figures and Tables

**Table 1:** Distribution of family and individual physical activity environment and characteristics of the study population stratified by sex groups of the 2019–2020 follow-up study. PA: Physical activity.

Variables	Total (n = 5008)	Boys (n = 2508)	Girls (n = 2500)	*p* Value
Residence				0.003
Urban	2754 (55.0)	1324 (52.8)	1430 (57.2)	
Rural	2254 (45.0)	1184 (47.2)	1070 (42.8)	
Incidence				<0.001
Overweight	723 (14.3)	438 (17.3)	285 (11.3)	
Obesity	573 (11.3)	309 (12.2)	264 (10.5)	
Transition				<0.001
Overweight	309 (6.2)	172 (6.9)	137 (5.5)	
Obesity	508 (10.1)	267 (10.7)	241 (9.6)	
Family and individual PA environment				<0.001
Unfavorable	2282 (45.6)	1080 (43.1)	1202 (48.1)	
favorable	2726 (54.4)	1428 (56.9)	1298 (51.9)	
Family PA environment				0.007
Unfavorable	1197 (23.9)	639 (25.5)	558 (22.3)	
favorable	3811 (76.1)	1869 (74.5)	1942 (77.7)	
Individual PA environment				<0.001
Unfavorable	2155 (43.0)	1005 (40.1)	1150 (46.0)	
favorable	2853 (57.0)	1503 (59.9)	1350 (54.0)	
Specific indicators of family PA environment				
Parents are very supportive of my participation in sports activities during my spare time	2097 (41.9)	1042 (41.6)	1055 (42.2)	0.03
Parents like to take part in sports activities in their leisure time	2411 (48.1)	1153 (46.0)	1258 (50.3)	0.003
Specific indicators of the individual PA environment				
I strongly agree that exercise makes me look better	1361 (27.2)	682 (27.2)	679 (27.1)	0.051
I strongly agree that exercise gives me more energy	2444 (48.8)	1263 (50.4)	1181 (47.2)	0.050
I strongly agree that exercise makes me feel happier	2335 (46.6)	1262 (50.4)	1073 (42.9)	<0.001
I strongly agree that exercise makes me find it more interesting	2212 (44.2)	1213 (48.4)	999 (40.0)	<0.001
I strongly agree that exercise enables me to make more friends	2002 (40.0)	1112 (44.4)	890 (35.6	<0.001
I strongly agree that exercise makes me stronger	2891 (57.7)	1603 (64.0)	1288 (51.5)	<0.001
I strongly agree that exercise makes me like myself more	1878 (37.5)	1011 (40.4)	867 (34.7)	<0.001
I strongly agree that exercise gives me a better body shape	2255 (45.0)	1131 (45.1)	1124 (45.0)	<0.001
I strongly agree that exercise makes me feel healthier	3361 (67.1)	1732 (69.1)	1629 (65.2)	<0.001

## Data Availability

The data presented in this study are available on request from the corresponding author due to privacy reasons.

## Abbreviations

The following abbreviations are used in this manuscript:

OW	Overweight
OB	Obesity
PA	Physical activity
OR	Odds ratio
CI	Confidence Interval
